# ROS-Responsive Fluorinated Oxalate Nanomedicine for Dual Chemiluminescence/^1^⁹F MRI Imaging and Targeted Drug Release

**DOI:** 10.3390/ijms26073304

**Published:** 2025-04-02

**Authors:** Anatoly Peshkov, Anel Urazaliyeva, Dariyana Saiduldinova, Kazbek Kulbergenov, Nasir Bala Alhassan, Almaz Beisenbayev, Yerkin Shabdan, Bauyrzhan Umbayev, Vsevolod Peshkov, Timur Sh. Atabaev, Timur Elebessov, Tri Thanh Pham, Chang-Keun Lim

**Affiliations:** 1 Department of Chemical and Materials Engineering, School of Engineering and Digital Sciences, Nazarbayev University, Astana 010000, Kazakhstan; anatolii.peshkov@nu.edu.kz (A.P.); anel.urazaliyeva@nu.edu.kz (A.U.); dariyana.saiduldinova@nu.edu.kz (D.S.); kazbek.kulbergenov@nu.edu.kz (K.K.); nasirbala.alhassan@nu.edu.kz (N.B.A.); almaz.beisenbayev@nu.edu.kz (A.B.); 2 National Laboratory Astana, Nazarbayev University, Astana 010000, Kazakhstan; bauyrzhan.umbayev@nu.edu.kz; 3 Department of Chemistry, School of Science and Humanities, Nazarbayev University, Astana 010000, Kazakhstan; vsevolod.peshkov@nu.edu.kz (V.P.); timur.atabaev@nu.edu.kz (T.S.A.); 4 Department of Biology, School of Science and Humanities, Nazarbayev University, Astana 010000, Kazakhstan; timur.elebessov@nu.edu.kz (T.E.); tri.pham@nu.edu.kz (T.T.P.)

**Keywords:** theranostics, nanomedicine, bioimaging, reactive oxygen species, chemiluminescence, ^19^F MRI, smart nanocarrier, cancer therapy

## Abstract

In this study, we developed a novel theranostic nanomedicine formulation that integrates multimodal imaging with controlled drug release in reactive oxygen species (ROS)-rich microenvironments. A fluorinated oxalate compound (FOC) was synthesized through a one-step condensation reaction between 1,1,1,3,3,3-hexafluoro-2-propanol and oxalyl chloride, characterized by ^1^H, ^13^C, and ^1^⁹F NMR spectroscopy. The FOC and luminophore-incorporated nanomedicine formulations reacted rapidly with hydrogen peroxide via the peroxyoxalate chemiluminescence (POCL) mechanism, producing strong chemiluminescence and inducing a notable 19-fold increase in ratiometric ^1^⁹F NMR signal upon conversion to fluorinated alcohol (FAH), demonstrating promising potential for high-contrast ^1^⁹F MRI in deep tissue. Following ROS stimulation, the chemical conversion from hydrophobic FOC to hydrophilic FAH led to the degradation of the nanomedicines, facilitating payload release. In vitro experiments with A-431 cancer cells under hypoxic conditions confirmed ROS-responsive drug release, evidenced by enhanced fluorescence from model luminophores. Additionally, doxorubicin-loaded FOC nanomedicines reduced cell viability to 32% under hypoxia while remaining non-toxic in normoxic conditions. These results indicate that FOC-based nanomedicine formulations provide a promising platform for combined chemiluminescence and ^1^⁹F MRI with targeted therapeutic efficacy in ROS-rich inflammatory and cancerous tissues.

## 1. Introduction

Recent advances in molecular biology and drug delivery have spurred the development of smart nanomedicines that can selectively activate therapeutic agents and diagnostic probes within targeted tissues, responding to disease-specific microenvironments and biomarkers. Biomarkers—tools that help elucidate disease prediction, etiology, diagnosis, progression, regression, and treatment outcomes—are central to the emerging field of theranostics, which integrates therapy and diagnosis [1].

Reactive oxygen species (ROS) are high-energy, oxygen-containing molecules that function as essential second messengers in cellular signaling. However, when produced in excess, ROS are implicated in a range of inflammatory conditions, including diabetes [2], neurodegenerative disorders [3], ischemia–reperfusion injury [4], impaired stem cell renewal [5], and cancer [6]. Accordingly, ROS serve as valuable biomarkers for theranostic strategies [7,8].

Fluorescent probes have traditionally been employed for ROS detection [9,10], but their utility is hampered by issues such as autofluorescence and limited excitation light penetration in vivo. In contrast, chemiluminescent probes—which generate light through intrinsic chemical reactions rather than external excitation—offer a promising alternative [11]. In particular, peroxyoxalate chemiluminescence (POCL), triggered by hydrogen peroxide (HP), has emerged as an attractive modality for in vivo imaging due to its high contrast and elimination of external light sources [12,13,14]. Nevertheless, the shallow imaging depth of optical probes remains a significant barrier for deep-tissue and whole-body applications.

To overcome this limitation, we have developed a multimodal imaging strategy that integrates POCL nanoparticles with a ROS-responsive clinical imaging modality. The POCL mechanism proceeds through a four-step process: (1) interaction between ROS and the oxalate moiety (–O–(CO)–(CO)–O–, which acts as the chemiluminescent fuel); (2) formation of the high-energy intermediate 1,2-dioxetanedione; (3) energy transfer from 1,2-dioxetanedione to a neighboring luminophore; and (4) emission of light from the excited luminophore. Here, we need to focus on the byproduct; the byproducts of the POCL reaction—such as alcohols and carbon dioxide (CO_2_)—can be leveraged for additional imaging modalities. In our previous work, we repurposed CO_2_ to generate microbubbles for the ultrasound imaging of inflammatory diseases [15].

**Figure 1:**
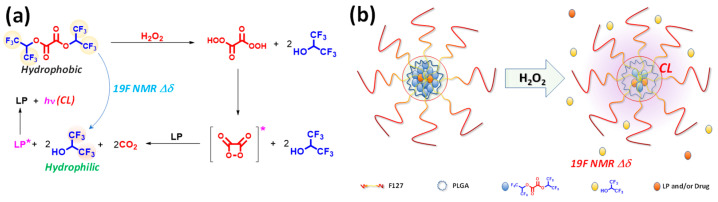
(**a**) Chemical reaction scheme depicting the peroxyoxalate chemiluminescence (POCL) reaction of bis(1,1,1,3,3,3-hexafluoropropan-2-yl) oxalate (FOC) with hydrogen peroxide (HP). (**b**) Schematic illustration of the ROS-responsive formulation of FOC nanomedicine (FOC NM), highlighting its dual functionality in targeted drug (and/or luminophore (LP)) release and multimodal imaging through chemiluminescence (CL) and ^1^⁹F MRI signal generation upon HP stimulation.

In this study, we introduce a novel theranostic nanomedicine formulation that simultaneously harnesses ROS-mediated multimodal imaging and controlled drug release. We synthesized a hydrophobic fluorinated oxalate compound, bis(1,1,1,3,3,3-hexafluoropropan-2-yl) oxalate (FOC), which reacts with hydrogen peroxide via the peroxyoxalate chemiluminescence (POCL) mechanism (Figure 1a). In our formulation, the intrinsic hydrophobicity of FOC drives its aggregation within the nanoparticle core alongside a luminophore, while Pluronic F-127 and PLGA enhance biocompatibility and structural stability.

In ROS-rich environments, the POCL reaction initiates the perhydrolysis of FOC, forming a high-energy 1,2-dioxetanedione intermediate that produces hydrophilic fluorinated alcohol (FAH, 1,1,1,3,3,3-hexafluoro-2-propanol) and carbon dioxide. The chemical energy released is transferred to adjacent luminophores, generating chemiluminescence (CL), while the conversion from FOC to FAH induces a distinct ^19^F NMR chemical shift, providing a bio-orthogonal imaging signal that complements conventional ^1^H MRI. Moreover, the hydrophilic transformation destabilizes the nanoparticle core, facilitating site-specific drug release within ROS-rich microenvironments typical of hypoxic cancer cells. Collectively, these results demonstrate the feasibility of FOC-based nanomedicine formulation as an integrated theranostic platform.

## 2. Results and Discussion

**Figure 2:**
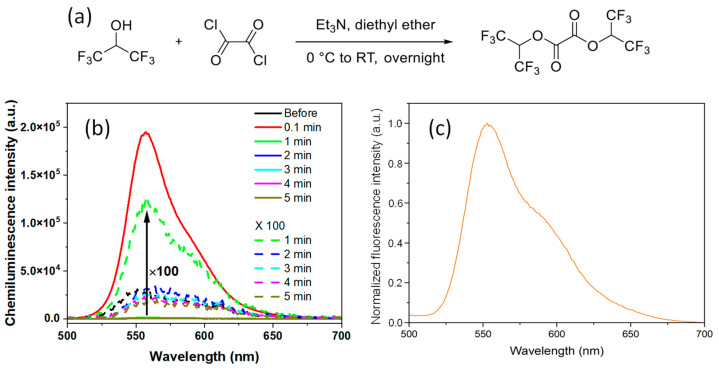
(**a**) Reaction scheme illustrating the one-step condensation between 1,1,1,3,3,3-hexafluoro-2-propanol and oxalyl chloride to yield bis(1,1,1,3,3,3-hexafluoropropan-2-yl) oxalate (FOC). (**b**) Time-dependent chemiluminescence (CL) spectra of an FOC/rubrene solution in tetrahydrofuran (THF) following the addition of 1 M hydrogen peroxide (HP). Dashed lines represent a 100-fold magnification of the corresponding original spectra, shown as solid lines. (**c**) Photoluminescence spectrum of rubrene solution in tetrahydrofuran (THF) upon excitation at 480 nm.

A novel ROS-responsive fluorinated oxalate compound, bis(1,1,1,3,3,3-hexafluoropropan-2-yl) oxalate (FOC), was designed and synthesized through a one-step condensation reaction between 1,1,1,3,3,3-hexafluoro-2-propanol and oxalyl chloride (Figure 2a). The purity and structure of FOC were confirmed by ^1^H, ^13^C, and ^1^⁹F NMR spectroscopy (Figures S1–S3, Supplementary Materials). Additionally, FOC demonstrated good solubility in common organic solvents, including dichloromethane (DCM), tetrahydrofuran (THF), and methanol, facilitating its incorporation into various nanoplatforms. The hydrogen peroxide (HP)-responsive chemiluminescent (CL) properties of FOC were investigated in THF. A solution containing 10 mg of FOC and 0.2 mg of rubrene was prepared, and upon adding 1 M HP, a luminescence peak at 560 nm was observed within 0.1 min without external photoexcitation (Figure 2b). The rapid decay in luminescence over time indicates swift perhydrolysis of FOC. This fast POCL reaction is attributed to the strong electron-withdrawing effect of the fluorine atoms, which enhances the nucleophilic attack of HP on the carbonyl groups of FOC. Notably, the CL spectra of the FOC-based nanomedicine (NM) were consistent with the fluorescence spectrum of the rubrene solution in THF (Figure 2c).

**Figure 3:**
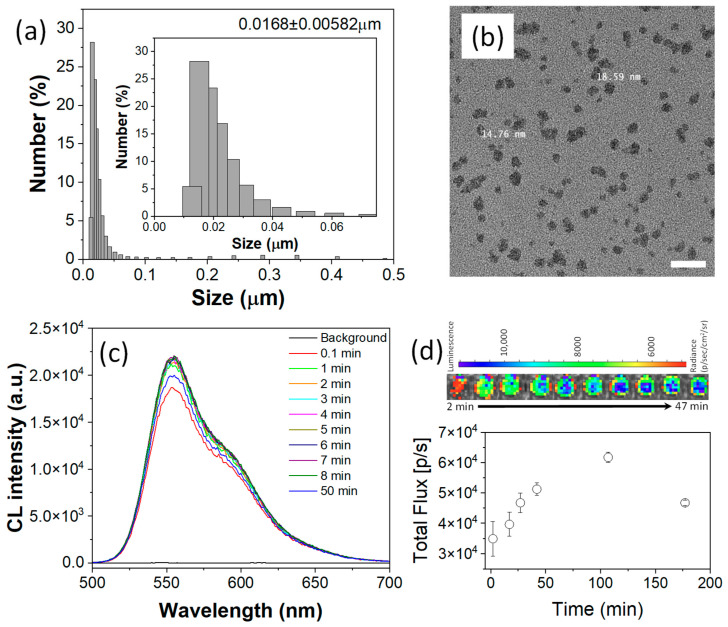
(**a**) Hydrodynamic size distribution of FOC nanomedicines (NMs) as determined by dynamic light scattering (DLS). The inset shows a magnified size distribution in 0 to 0.075 μm. (**b**) Transmission electron microscopy (TEM) image of FOC NMs, confirming their uniform morphology. Scale bar shows 50 nm. (**c**) Time-dependent chemiluminescence (CL) spectra of the FOC NM suspension, recorded before and after the addition of 10 μM hydrogen peroxide (HP). (**d**) Representative time-dependent CL images (upper panel) and corresponding CL intensity profiles (lower panel) of the FOC NM suspension, illustrating the response to 10 μM HP.

To demonstrate the theranostic potential of FOC, a biocompatible nanomedicine (NM) was developed. The nanomedicine was constructed using an amphiphilic polymer matrix of Pluronic F-127 to ensure water dispersibility and biocompatibility, while PLGA was employed to enhance nanostructural stability. Within the F-127/PLGA micellar matrix, FOC, as a ROS-responsive physical and chemical modifier, was co-encapsulated with Bis(2,4,5-trichloro-6-(pentyloxycarbonyl)phenyl)oxalate (CPPO) (a well-established CL fuel) as a co-fuel for stronger CL and rubrene as the CL emitter. An oil-in-water nanoemulsion method followed by solvent evaporation yielded an aqueous suspension of FOC NMs with an average hydrodynamic diameter of approximately 20 nm and a narrow size distribution (Figure 3a), making it suitable for systemic circulation [16]. Transmission electron microscopy (TEM) images (Figure 3b) further confirmed these dimensions.

The CL performance of the FOC NM was assessed by adding HP to its aqueous suspension. Even at a low concentration of HP (10 μM) [17], a consistent CL signal was observed for up to 50 min (Figure 3c). Analyzing the area under the curve indicated an approximately 560-fold signal enhancement within 0.1 min after HP addition, ensuring an ultra-high contrast-to-noise ratio for bioimaging applications. Furthermore, the close match between the CL spectrum of the NM suspension and that of the FOC/rubrene solution suggests that the spectral region can be adjusted by selecting appropriate colocalized luminophores.

The CL imaging capability was further demonstrated in an IVIS SpectrumCT system using 10 μM HP (Figure 3d). Despite the relatively low HP concentration, the high sensitivity of the cooled CCD enabled the detection of a CL signal that persisted for over 3 h, which is appropriate for in vivo imaging applications. Time-dependent profiles indicated that the CL signal continued to increase up to 107 min post-HP addition. This prolonged signal likely reflects the heterogeneous reaction kinetics within the nanostructured system—where the POCL reaction is initiated at the nanoparticle surface and gradually propagates inward—and correlates with particle degradation driven by the conversion of hydrophobic FOC into hydrophilic fluorinated alcohol (FAH), a process essential for ROS-responsive drug release.

Multimodal imaging is achieved by combining complementary modalities to overcome individual limitations [18]. Although CL imaging offers a simple real-time setup, its tissue penetration is inherently limited by light absorption and scattering. In contrast, magnetic resonance imaging (MRI) provides high-resolution, whole-body imaging via the ^1^H signal. By leveraging the bioorthogonality of ^1^⁹F nuclei, which offer distinct resonance properties compared to ^1^H, remarkably high contrast in MRI can be achieved [19,20].

FOC contains six fluorine atoms, making it inherently suitable as an ^1^⁹F MRI contrast agent. More importantly, the perhydrolysis of FOC with HP induces a measurable ^1^⁹F NMR chemical shift. In FOC, the fluorine atoms reside in an electron-deficient environment due to the electron-withdrawing oxalate group (–O–(CO)–(CO)–O–), resulting in a higher resonance frequency. Conversion to FAH ((CF_3_)_2_–CH–OH) increases the electron density around the fluorine atoms, thereby lowering the resonance frequency. This ROS-responsive chemical shift effectively decouples the biodistribution of the nanomedicine from the ROS-rich regions of inflammatory diseases.

**Figure 4:**
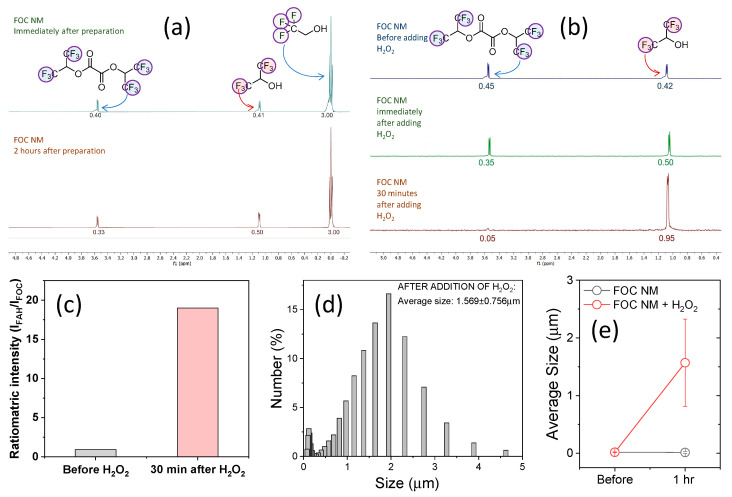
(**a**) ^19^F NMR spectra of the FOC NM suspension in water, recorded immediately after preparation and 2 h later. (**b**) ^19^F NMR spectra of the FOC NM suspension in water, obtained immediately before, immediately after, and 30 min after the addition of 10 μM hydrogen peroxide (HP). In (**a**,**b**), 2,2,2-trifluoroethanol was used as the ^19^F NMR reference (chemical shift set to 0 ppm). (**c**) Ratiometric signal intensity (I_FAH_/I_FOC_) derived from ^19^F NMR spectra of FOC NM before and 30 min after 10 μM HP addition. (**d**) Size distribution of FOC NMs measured by dynamic light scattering (DLS) 1 h after treatment with 10 μM HP. (**e**) Average particle size ± standard error of FOC NMs measured before and 1 h after treatment with HP (FOC NM + HP) or water (FOC NM control).

The ^1^⁹F NMR imaging capability was demonstrated by comparing the spectra of FOC NMs with and without HP. Using 2,2,2-trifluoroethanol as an internal reference (set at zero ppm), the spectra revealed an FOC peak at 3.52 ppm and an FAH peak at 1.08 ppm immediately after NM preparation (Figure 4a). The nearly equal integration of these peaks indicated that approximately 50% of FOC had undergone hydrolysis in deionized water. Two hours later, the FOC signal decreased by 18% while the FAH signal increased by about 22%, reflecting moderate spontaneous hydrolysis and indicating that the nanostructure remains reasonably stable in aqueous suspension.

In contrast, upon the addition of HP to the FOC NM suspension, the FOC peak decreased by 22%, and the FAH peak increased by 18% immediately, with further changes (an 89% decrease in FOC and a 126% increase in FAH) observed after 30 min (Figure 4b). The resulting chemical shift difference (Δδ = 2.44 ppm, from 3.52 ppm for FOC to 1.08 ppm for FAH) permits selective imaging of FAH distribution using Larmor frequency-selective ^19^F MRI techniques [21,22,23], thereby enhancing contrast in regions of inflammatory disease.

Additionally, the aggregation of hydrophobic FOC molecules typically results in a shortened spin–spin (T_2_) relaxation time and a suppressed ^1^⁹F MRI signal. However, the POCL reaction converts FOC into hydrophilic FAH, leading to disassembly of the hydrophobic core, prolonged relaxation times, and an enhanced ^1^⁹F MRI signal [24,25]. This is reflected in the superior signal enhancement of the FAH peaks under both water- (22% increase over 2 h) and HP-treated conditions (126% increase after 30 min), compared to the corresponding decrease in FOC peaks. Although the initial FAH signal enhancement is modest—likely due to a gradual release process—the overall change can be further amplified by ratiometric imaging, which involves dividing the FAH signal by the FOC signal. As shown in Figure 4c, the ratiometric intensity (I_FAH_/I_FOC_) increased 19-fold 30 min after HP addition, far exceeding the 2.26-fold contrast obtained by a simple FAH signal comparison. This ratiometric approach, analogous to logarithmic digital subtraction in contrast-enhanced angiography [26], yields significant contrast for selectively imaging FAH, thereby mapping ROS-rich microenvironments in vivo.

The hydrophilicity conversion from FOC to FAH not only enhances imaging contrast but also triggers nanoparticle degradation, a key feature for controlled drug release. This ROS-responsive degradation was monitored using dynamic light scattering (DLS). Initially, the FOC NMs exhibited an average hydrodynamic diameter of approximately 17 nm (±5.8 nm) (Figure 3a). When the NMs’ suspension was divided and one portion treated with hydrogen peroxide (HP), DLS measurements taken 1 h later revealed an abrupt increase in particle size to around 1.6 μm (±756 nm) (Figure 4d,e), indicating nanostructure degradation. In contrast, the control suspension maintained a stable size (14.2–18.6 nm over 1 h), confirming that ROS specifically triggers the degradation. These results underscore the potential of FOC NMs for targeted drug delivery and site-specific release in ROS-rich inflammatory regions.

**Figure 5:**
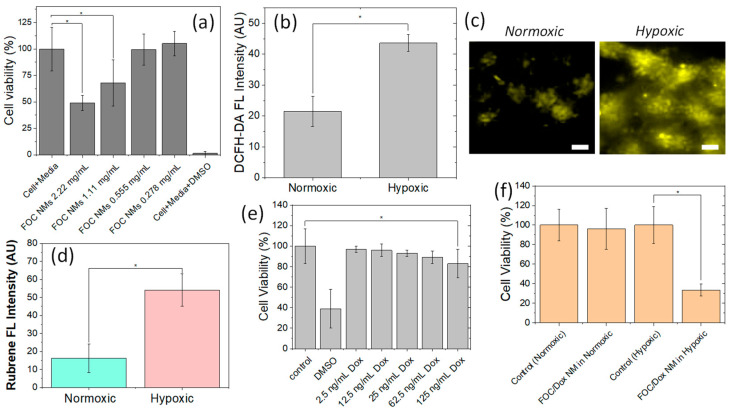
(**a**) Cytotoxicity of FOC nanomedicines (NMs) in A-431 cells, assessed using the Presto Blue cell viability assay. The concentrations represent the levels of FOC NMs in the cell culture wells. Data are shown as mean ± standard deviation, and statistical significance was determined using one-way ANOVA (* *p* < 0.05). (**b**) DCFH-DA fluorescence intensity of A-431 cells under hypoxic compared to normoxic conditions (in an anaerobic bag), evaluated with a Vario Skan microplate reader. Statistical significance was assessed using one-way ANOVA (* *p* < 0.05). (**c**) Representative fluorescence microscopy images of A-431 cells treated with FOC NMs under normoxic and hypoxic conditions; scale bars represent 10 μm. (**d**) Quantitative analysis of rubrene fluorescence intensity in A-431 cells treated with FOC NMs under hypoxic and normoxic conditions, indicating ROS-responsive rubrene release. Statistical significance was assessed using one-way ANOVA (* *p* < 0.05). (**e**) Cytotoxicity of FOC/Dox NMs as a function of doxorubicin loading, determined by the Presto Blue cell viability assay. Statistical significance was assessed using one-way ANOVA (* *p* < 0.05). (**f**) Viability of A-431 cells treated with FOC/Dox NMs (0.555 mg/mL NMs including 12.5 ng/mL Dox) under hypoxic versus normoxic conditions; statistical significance was assessed using one-way ANOVA (* *p* < 0.05).

To demonstrate the application of FOC NMs in cancer cells, we first assessed their cytotoxicity to determine a non-toxic dose for further studies. A-431 cells (a human epidermoid carcinoma cell line) were incubated with FOC NMs at concentrations of 2.22, 1.11, 0.555, and 0.278 mg/mL for 24 h, and cell viability was measured using the Presto Blue assay. As shown in Figure 5a, concentrations above 1.11 mg/mL exhibited significant toxicity, whereas concentrations below 0.555 mg/mL were non-toxic under the experimental conditions. Based on these data, subsequent experiments were conducted using the 0.555 mg/mL concentration of FOC NMs.

Hypoxia is a defining characteristic of the cancer tissue microenvironment, marked by insufficient oxygen levels that disrupt homeostasis. Reports indicate that cancer cells, under hypoxic conditions, process intracellular oxygen through mechanisms involving mitochondrial respiratory complex III [27]. To evaluate ROS-responsive drug release from FOC/Dox NMs, hypoxic conditions were established in A-431 cells by incubating them in an anaerobic bag for 12 h. The effectiveness of hypoxia induction was confirmed using the fluorescent ROS probe dichloro-dihydro-fluorescein diacetate (DCFH-DA), which showed that hypoxic cells exhibited approximately twice the fluorescence intensity of normoxic cells (Figure 5b).

A fluorescence-based visualization method was used to demonstrate ROS-responsive drug release. In the densely packed nanocarrier, the fluorescence (FL) of organic luminophores is quenched by concentration quenching. However, upon release and subsequent dilution in the cytosol, the FL is enhanced. Using rubrene molecules in FOC NMs as a model, we observed that after treating A-431 cells with a 0.555 mg/mL suspension of FOC NMs for 12 h, cells under hypoxic conditions (anaerobic bag) showed a discernible increase in FL compared to normoxic cells (Figure 5c). Quantitative analysis revealed that FL intensity in hypoxic cells was over 3.5-fold higher than in normoxic cells (Figure 5d), indicating efficient release of rubrene following ROS-responsive degradation of the NMs under hypoxic conditions. In contrast, under normoxic conditions, the FOC NMs stably retained the luminophores within their cores.

To further evaluate the ROS-responsive therapeutic efficacy of FOC NMs, doxorubicin (Dox) was loaded into the nanocarriers. Initially, we established the maximum non-toxic dosage of Dox within FOC NMs via cytotoxicity assays. The goal of this activatable (smart) nanocarrier is to minimize damage to healthy tissues during chemotherapy while enhancing therapeutic efficacy at the target site through selective drug release. Dox concentrations of 0.1 μg, 0.5 μg, 1 μg, 2.5 μg, and 5 μg (2.5 ng/mL, 12.5 ng/mL, 25 ng/mL, 62.5 ng/mL and 125 ng/mL in cell-culturing solution, respectively) were incorporated into the precursor solution for FOC NMs, resulting in the preparation of FOC/Dox NMs. The FOC/Dox NMs were added to cell-culturing solutions without purifications to remove unloaded Dox. When 0.555 mg/mL FOC/Dox NMs were incubated with A-431 cells for 24 h, minimal toxicity was observed for formulations containing less than 0.5 μg (12.5 ng/mL in cell-culturing solution) of Dox (Figure 5e). Thus, FOC/Dox NMs containing 12.5 ng/mL Dox were selected for further evaluation, as they do not elicit significant cytotoxicity at normal ROS levels but can potentially demonstrate enhanced efficacy under abnormally high ROS conditions.

Finally, we compared the therapeutic efficacy of FOC/Dox NMs under hypoxic and normoxic conditions. A-431 cells were cultured in two separate 96-well plates and treated with 0.555 mg/mL FOC/Dox NMs, including 12.5 ng/mL Dox. One plate was incubated in an anaerobic bag (hypoxic conditions) and the other under ambient air (normoxic conditions) for 12 h. Subsequent Presto Blue cell viability assays revealed that hypoxic A-431 cells treated with FOC/Dox NMs exhibited a significantly reduced viability of 32%, whereas normoxic cells maintained higher viability (Figure 5f). These results strongly support the concept that FOC/Dox NMs can effectively target and reduce hypoxic cancer tissue margins through ROS-responsive drug release.

## 3. Materials and Methods

### 3.1. Materials

1,1,1,3,3,3-Hexafluoro-2-propanol, anhydrous diethyl ether, and anhydrous triethylamine were purchased from Sigma-Aldrich (St. Louis, MO, USA). Pluronic F-127, poly(D,L-lactide-co-glycolide) (PLGA, ester terminated, Mw 50,000–75,000), rubrene (sublimed grade), doxorubicin hydrochloride, dichloromethane (anhydrous), and hydrogen peroxide were also obtained from Sigma-Aldrich. Bis[3,4,6-trichloro-2-(pentyloxycarbonyl)phenyl] oxalate was purchased from TCI (Tokyo, Japan). Deionized water, fetal bovine serum (FBS), trypsin, Dulbecco’s modified Eagle’s medium (DMEM), streptomycin, and Presto Blue cell viability kits were procured from Gibco, Thermo Fisher Scientific (Waltham, MA, USA). The fluorescent probe 2′,7′-dichlorodihydrofluorescein diacetate (DCFH-DA) and phosphate-buffered saline (PBS) were purchased from Santa Cruz Biotechnology (Dallas, TX, USA). A-431 cells were obtained from ATCC Cell Bank (Manassas, VI, USA).

### 3.2. Synthesis of Fluorinated Oxalate Compound (FOC) 

Bis(1,1,1,3,3,3-hexafluoropropan-2-yl) oxalate (FOC) was synthesized as a hydrogen peroxide-responsive peroxalate compound. In a Schlenk tube cooled in an ice bath, 1.75 mL (20.00 mmol) of oxalyl chloride was mixed with 40 mL of anhydrous diethyl ether under a nitrogen atmosphere. Next, 4.50 mL (42.8 mmol) of 1,1,1,3,3,3-hexafluoro-2-propanol was added, followed by dropwise addition of a solution containing 10 mL of anhydrous diethyl ether and 5.5 mL (40.15 mmol) of triethylamine. A heavy white precipitate formed, and the reaction mixture was stirred overnight as the ice melted. The mixture was then filtered via a Büchner funnel, and the filtrate was concentrated using a rotary evaporator at 650 mbar and 41 °C for 7 min to remove most of the ether. The product was distilled, and the fraction collected at 126–127 °C yielded 2.386 g of clear oil. Structural confirmation was obtained by ^1^H, ^13^C, and ^1^⁹F NMR using a JNM-ECA FT NMR spectrometer operating at 500 MHz for ^1^H. (^1^H NMR (500 MHz, CDCl_3_): δ 5.81, heptet, 2H; ^13^C NMR (125 MHz, CDCl_3_): δ 152.35 (singlet, –O–(**C**O)–(**C**O)–O–), δ 120 (quartet, –**C**F_3_), δ 68.9 (quintet, –**C**(CF_3_)_2_); ^1^⁹F NMR (470 MHz, CDCl_3_): δ −72.90 (singlet, –C**F**_3_).

### 3.3. Preparation of FOC NMs

FOC NMs were prepared via an oil-in-water nanoemulsion method. A mixture containing 2 mg FOC, 2 mg bis[3,4,6-trichloro-2-(pentyloxycarbonyl)phenyl] oxalate (CPPO), 0.04 mg rubrene, and 0.4 mg PLGA was dissolved in 500 μL of dichloromethane. This organic phase was slowly added to 2 mL of deionized water containing 40 mg of Pluronic F-127 while sonicating (50% amplitude; 3 s on, 1 s off) for 2 min. The dichloromethane was then evaporated at room temperature using a rotary evaporator, yielding an aqueous suspension of FOC NMs at 22.2 mg/mL. For FOC/Dox NMs, predetermined amounts of doxorubicin (0.1, 0.5, 1, 2.5, or 5 μg) were added to the dichloromethane solution before nanoemulsion formation.

### 3.4. Characterization of NMs

Dynamic light scattering (DLS) measurements were performed using theNanotrac Wave II (Microtrac Inc., York, PA, USA) (temperature: 24.2 ± 0.1 °C) to determine hydrodynamic size distributions. Transmission electron microscopy (TEM) images were acquired on JEM-1400Plus Electron Microscope (JEOL Ltd., Tokyo, Japan) at 120 kV. Samples for TEM were prepared by depositing a drop onto a grid and blotting away excess solvent with filter paper.

### 3.5. Chemiluminescence and Fluorescence Measurements

Chemiluminescence (CL) and fluorescence spectra were recorded using an FLS 1000 Photoluminescence Spectrometer (Edinburgh Instruments, Livingston, UK) equipped with a xenon lamp and a PMT-900 detector. For FOC/rubrene CL measurements, 10 mg FOC and 0.2 mg rubrene were dissolved in 1 mL THF, followed by addition of 1 mL of 2 M HP in THF. CL spectra were acquired immediately and then every minute for 5 min without excitation light. For FOC NM suspensions, 1 mL of a 22.2 mg/mL NM sample was mixed with 1 mL of a 20 μM hydrogen peroxide solution, and CL spectra were recorded similarly. Fluorescence spectra of a 10 μM rubrene solution in THF were recorded under 480 nm excitation. Time-dependent CL imaging of FOC NMs was performed using an IVIS SpectrumCT (PerkinElmer, Waltham, MA, USA). In these experiments, 100 μL of a 22.4 mg/mL FOC NM suspension was mixed with 100 μL of a 20 μM HP solution in 96-well plates, and images were acquired immediately (exposure: 1 min; f/stop: 1; binning: 8; optical filter: 560 nm).

### 3.6. Cytotoxicity Tests for Evaluating Material Toxicity and Drug Efficacy

The Presto Blue cell viability assay was used to assess the cytotoxicity of FOC NMs and FOC/Dox NMs in A-431 cells. Cells were seeded at 5000 cells per well in a 96-well plate using DMEM supplemented with 10% FBS, 1% penicillin/streptomycin, and 1% nonessential amino acids, and incubated overnight at 37 °C in a humidified atmosphere (20% O₂, 5% CO_2_). For FOC NM toxicity testing, 5 μL of NM suspensions at concentrations of 44.4, 22.2, 11.1, or 5.55 mg/mL was added to 95 μL of cell culture medium. After 24 h, cells were washed with PBS and incubated with 10 μL Presto Blue (Thermo Fisher Scientific, Waltham, MA, USA) for 20 min (protected from light), and fluorescence was measured on a Varioskan LUX Multimode microplate reader (Thermo Fisher Scientific, Waltham, MA, USA) (λ_ex_: 560 nm, λ_em_: 590 nm). For FOC/Dox NM tests, 5 μL of a 11.1 mg/mL NM suspension containing varying amounts of Dox (0.1, 0.5, 1, 2.5, or 5 μg) was added to cell culture wells (95 μL of medium), followed by the same procedure. For FOC/Dox NMs’ ROS-responsive drug efficacy evaluation, hypoxic conditions were induced using an anaerobic bag; A-431 cells were seeded in two 96-well plates, treated with 5 μL of 11.1 mg/mL FOC/Dox NMs (0.5 μg Dox) in cell culture wells (95 μL medium), and incubated for 12 h under hypoxic (anaerobic bag) or normoxic conditions before performing the Presto Blue assay.

### 3.7. DCFH-DA ROS Assay for Hypoxic/Normoxic A-431 Cells

Five thousand A-431 cells were seeded per well in two 96-well plates and cultured in DMEM for 12 h at 37 °C. One plate was incubated in an anaerobic bag (hypoxic), while the other remained under normoxic conditions (20% O_2_, 5% CO_2_) for 24 h. A 10 mM stock solution of DCFH-DA was prepared by dissolving 4.85 mg in 1 mL of DMSO and, immediately before the assay, diluted in pre-warmed DMEM to obtain a 10 μM working solution. After aspirating the medium and washing with DMEM, 100 μL of the DCFH-DA solution was added to each well and incubated at 37 °C for 30 min. The wells were then washed with DMEM and PBS, and 100 μL PBS was added for fluorescence measurement using a Varioskan LUX Multimode microplate reader (λ_ex_: 485 nm, λ_em_: 535 nm).

### 3.8. Fluorescent Imaging of Intracellular Drug Release

A-431 cells were seeded at 5000 cells per well in 8-well Ibidi plates and incubated overnight at 37 °C in a humidified atmosphere (20% O_2_, 5% CO_2_). The medium was then replaced, and 5 μL of a 11.1 mg/mL FOC NM suspension was added to each cell culture well (95 μL of cell culture medium). One plate was incubated under hypoxic conditions (anaerobic bag) and the other under normoxic conditions for 24 h. Prior to imaging, the wells were washed twice with DMEM. Fluorescence imaging was performed using the Leica Thunder Imager and Steadycon Microscope.

### 3.9. Data Analysis

All data are presented as a mean ± standard deviation. Statistical analyses were conducted using ANOVA and Student’s t-test, with significance set at *p* < 0.05. For image analysis, fluorescence and chemiluminescence intensities from images were quantified through region of interest (ROI) analysis.

## 4. Conclusions

In summary, we have developed a novel ROS-responsive theranostic nanomedicine formulation based on a fluorinated oxalate compound (FOC) that effectively integrates multimodal imaging with controlled drug release. The unique design of FOC enables rapid perhydrolysis via the peroxyoxalate chemiluminescence (POCL) mechanism upon exposure to hydrogen peroxide, generating a chemiluminescent signal and inducing a measurable ^1^⁹F NMR chemical shift. This dual imaging capability not only enhances the contrast for in vivo detection but also circumvents the depth limitations inherent to conventional optical imaging techniques.

The incorporation of FOC into a biocompatible nanocarrier system—with Pluronic F-127 and PLGA ensuring stability and dispersibility—resulted in nanomedicines of appropriate size for systemic circulation. Under ROS-rich conditions, the hydrophobic-to-hydrophilic conversion of FOC to FAH triggered nanoparticle degradation, effectively releasing encapsulated payloads. This ROS-responsive drug release was validated using both model (rubrene) and therapeutic (doxorubicin) agents, with cytotoxicity studies in A-431 cancer cells demonstrating selective efficacy under hypoxic (ROS-rich) conditions.

Collectively, these findings underscore the significant potential of FOC-based nanomedicine formulations as versatile theranostic agents for targeted imaging and treatment of inflammatory diseases and cancers characterized by elevated ROS levels. Future studies will focus on optimizing this platform for in vivo applications, further advancing the integration of multimodal imaging with controlled drug delivery in precision medicine.

## Data Availability

Data are contained within the article and Supplementary Materials.

## Supplementary Materials

The following supporting information can be downloaded at: https://www.mdpi.com/article/10.3390/ijms26073304/s1.
