# Supplementary material for: ROS-Responsive Fluorinated Oxalate Nanomedicine for Dual Chemiluminescence/1⁹F MRI Imaging and Targeted Drug Release
Source: Int J Mol Sci. 2025 Apr 2;26(7):3304. doi: 10.3390/ijms26073304 (PMC11989819; doi:10.3390/ijms26073304)
Supplement: Supplementary file 1 [file ijms-26-03304-s001.zip › ijms-3498624-supplementary.pdf]

ROS-Responsive Fluorinated Oxalate Nanomedicine for Dual Chemiluminescence/^{19}F MRI Imaging and Targeted Drug Release

Ayaulym Abilova[1,+], Anatoly Peshkov[1,+], Anel Urazaliyeva[1,+], Dariyana Saiduldinova[1], Kazbek Kulbergenov[1], Nasir Bala Alhassan[1], Almaz Beisenbayev[1,2], Yerkin Shabdan[1,2,*], Bauyrzhan Umbayev[2], Vsevolod Peshkov[3], Timur Atabaev[3], Tri Pham[4], Chang-Keun Lim[1,*]

1 Department of Chemical and Materials Engineering, School of Engineering and Digital Sciences, Nazarbayev University, Astana 010000, Kazakhstan; ayaulym.abilova@alumni.nu.edu.kz (A.A.); anatolii.peshkov@nu.edu.kz (A.P.); anel.urazaliyeva@nu.edu.kz (A.U.); dariyana.saiduldinova@nu.edu.kz (D.S.); kazbek.kulbergenov@nu.edu.kz (K.K.); nasirbala.alhassan@nu.edu.kz (N.B.A.); almaz.beisenbayev@nu.edu.kz (A.B.)

2 National Laboratory Astana, Nazarbayev University, Astana 010000, Kazakhstan; bauyrzhan.umbayev@nu.edu.kz (B.U.)

3 Department of Chemistry, School of Science and Humanities, Nazarbayev University, Astana 010000, Kazakhstan; vsevolod.peshkov@nu.edu.kz (V.P.)

4 Department of Biology, School of Science and Humanities, Nazarbayev University, Astana 010000, Kazakhstan; tri.pham@nu.edu.kz (T.P.)

+ These authors contributed equally to this work.

* Correspondence: changkeun.lim@nu.edu.kz (C.-K.L); yerkin.shabdan@nu.edu.kz (Y.S.)

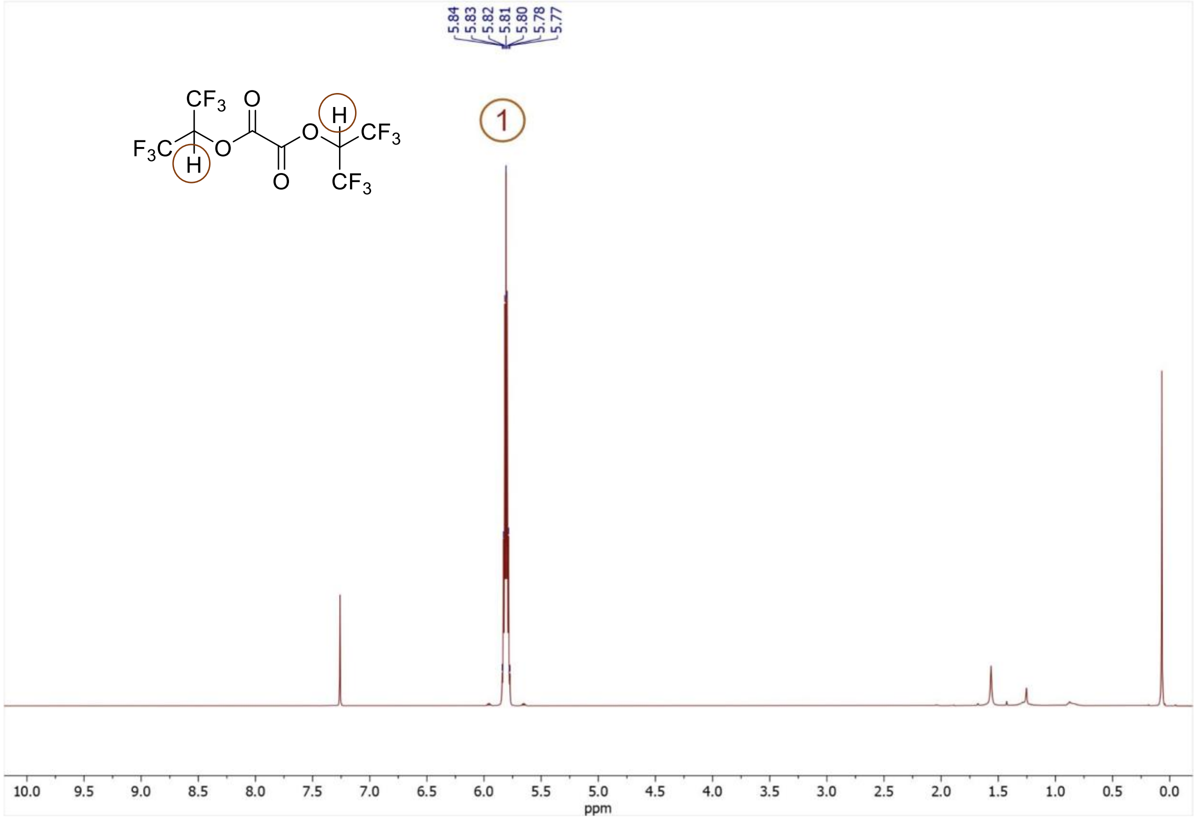

Figure S1. ^{1}H NMR (500 MHz, $CDCl_3$) spectrum of FOC

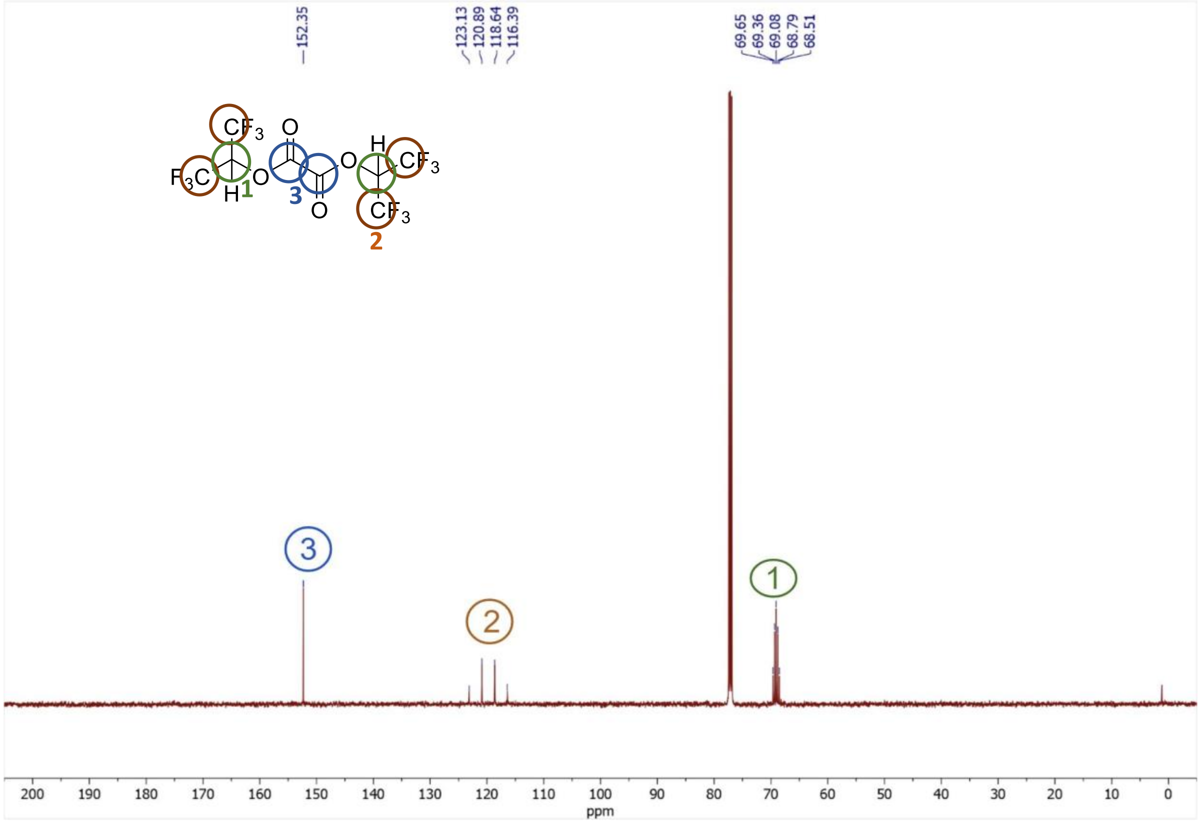

Figure S2. ^{13}C NMR (125 MHz, $CDCl_3$) spectrum of FOC

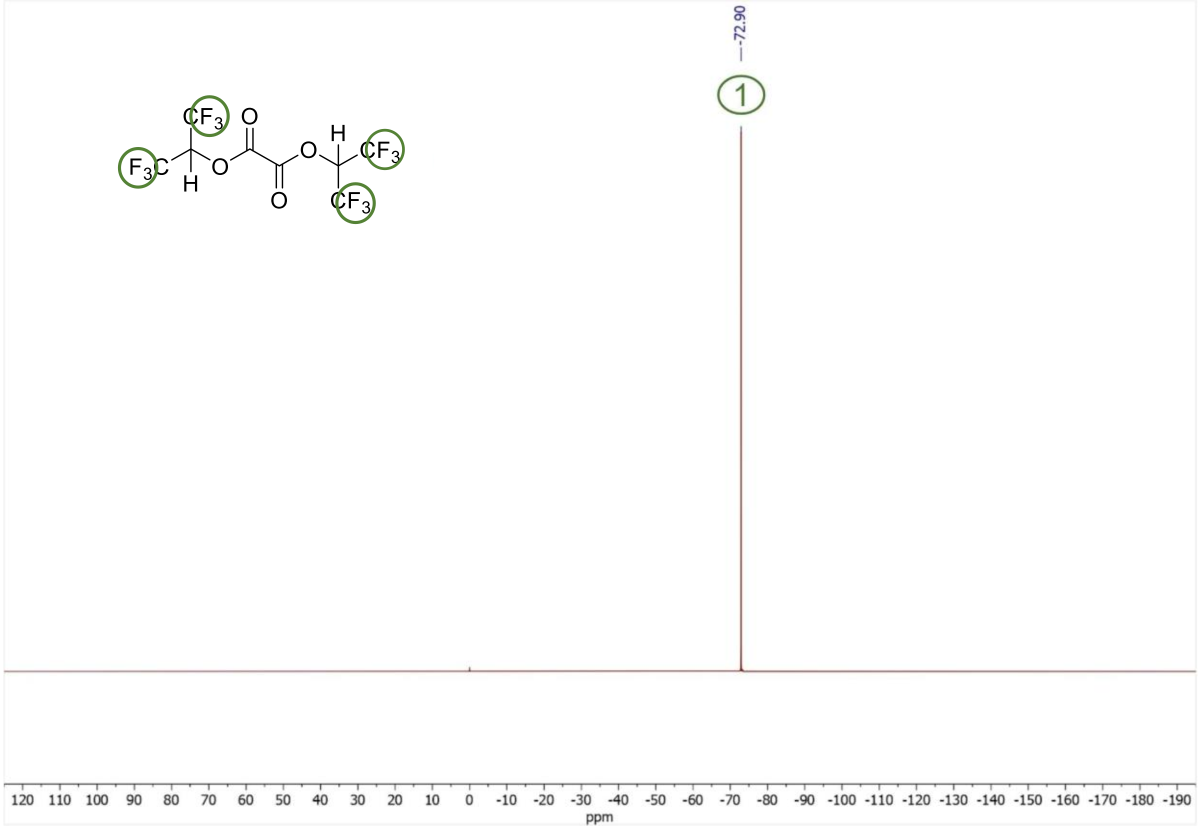

Figure S3. ^{19}F NMR (470 MHz, $CDCl_3$) spectrum of FOC
